# Prevalence and molecular characterization of ESBL-producing *Enterobacteriaceae* in Egypt: a systematic review and meta-analysis of hospital and community-acquired infections

**DOI:** 10.1186/s13756-024-01497-z

**Published:** 2024-12-05

**Authors:** Ahmed Azzam, Heba Khaled, Dareen Samer, Wedad M. Nageeb

**Affiliations:** 1 Department of Microbiology and Immunology, Faculty of Pharmacy, Helwan University, Cairo, Egypt; 2 Department of Biochemistry, Faculty of Pharmacy, Cairo University, Cairo, Egypt; 3 Department of clinical laboratory sciences, College of Applied Medical Scienes, King Khalid University, Abha, Saudi Arabia; 4 Medical Microbiology and Immunology Department, Faculty of Medicine, Suez Canal University, Ismailia, Egypt

**Keywords:** *E. Coli*, *K. pneumoniae*, Enterobacterales, *Enterobacteriaceae*, ESBL, ESBL-PE, *bla*_CTX−M_, Egypt, Meta-analysis

## Abstract

**Background:**

ESBL-producing *Enterobacteriaceae* (ESBL-PE) represent a significant global health threat. In response to this growing concern and the lack of a surveillance system for ESBL-PE infections in Egypt, we conducted this meta-analysis. In this study, we aimed to quantify the prevalence of ESBL-PE based on the source of infection and characterize their molecular dissemination. Additionally, we sought to uncover temporal trends to assess the spread of ESBL-PE over time.

**Methods:**

A comprehensive literature search was conducted in PubMed, Scopus, Google Scholar, Web of Science, and the Egyptian Knowledge Bank to identify studies that: (1) report the prevalence of ESBL-PE in Egypt; (2) use valid detection methods; (3) involve clinical specimens; and (4) were published between 2010 and 2024. The quality of the included studies was evaluated using the “Joanna Briggs Institute Critical Appraisal Checklist”. Meta-analysis was performed using the R meta package, reporting pooled prevalence with 95% confidence intervals (CI) via a random effects model.

**Results:**

This meta-analysis included 34 studies with 4,528 isolates, spanning 2007 to 2023. The overall prevalence of ESBL-PE in Egypt was 60% (95% CI: 54–65). The leave-one-out meta-analysis demonstrated the absence of influential outliers and Egger’s test indicated no evidence of publication bias (*P* = 0.25). The prevalence of ESBL-PE was 62% (95% CI: 55–68) in nosocomial infections and 65% (95% CI: 52–75) in community-acquired infections, with no statistically significant difference (*P* = 0.68). The prevalence of ESBL producers in *E. coli* (64%) and *K. pneumoniae* (63%) is higher than in *Proteus mirabilis* (46%) (*P* = 0.06). Temporal analysis showed a stable ESBL prevalence over time. Moreover, in phenotypically confirmed ESBL-producing, *E. coli* harboring *bla*_CTX−M_ was most prevalent (73%), followed by *bla*_TEM_ (60%) and *bla*_SHV_ (22%), with significant differences (*P* < 0.01). Subsequent analysis identified *bla*_CTX−M−15_ as the predominant variant of the *bla*_CTX−M_ gene.

**Conclusions:**

The prevalence of ESBL-PE in Egypt is alarmingly high at 60%. The observed high rates in both hospital and community-acquired infections underscore the need for public health strategies targeting both settings. One limitation of this study is the high heterogeneity, which partly attributed to regional and institutional variations in antibiotic use and stewardship practices.

**Supplementary Information:**

The online version contains supplementary material available at 10.1186/s13756-024-01497-z.

## Introduction

Antimicrobial resistance is emerging as an increasingly critical global health threat. Predictive statistical models estimate that bacterial antimicrobial resistance (AMR) directly accounted for approximately 1.27 million deaths worldwide in 2019 [1]. It is projected that by 2050, AMR could result in up to 10 million deaths annually [2]. In 2017, WHO published its first list of antibiotic-resistant bacteria that pose the greatest threat to human health, aiming to guide research and development of new antibiotics to combat rising AMR [3]. Among the pathogens on this list is ESBL-producing *Enterobacteriaceae* (ESBL-PE), classified as priority 1 (critical) [3]. Patients with ESBL-PE infections exhibit an unfavorable prognosis, marked by elevated mortality rates, prolonged durations of hospitalization, and diminished clinical and microbiological response outcomes [4–6].

Extended-spectrum β-lactamases (ESBLs) are enzymes produced by certain bacteria that can degrade a broad spectrum of β-lactam antibiotics, including penicillins, most cephalosporins, and aztreonam [7]. The most commonly used method for classifying beta-lactamases is the Ambler classification system, which categorizes these enzymes based on their amino acid sequence homology [8]. This classification divides beta-lactamases into four classes: A, B, C, and D. Classes A, C, and D are serine-β-lactamases (SBLs), which use a serine residue at their active site to hydrolyze the β-lactam ring of antibiotics [8]. In contrast, class B consists of metallo-β-lactamases (MBLs), which require zinc at their active sites to catalyze the breakdown of β-lactam antibiotics [8]. ESBLs are primarily classified under Ambler class A [8, 9]. The most frequently identified ESBLs in Ambler class A include the TEM, SHV, and CTX-M enzyme families, which are encoded by the *bla*_*TEM*_, *bla*_*SHV*_, and *bla*_*CTX−M*_ genes, respectively [9]. Carbapenems are frequently used to treat severe infections caused by ESBL-PE, but rising resistance to these antibiotics is becoming a concern. As the dependency on this vital class of drugs intensifies, the risk of carbapenem-resistant Enterobacterales correspondingly escalates [7].

The prevalence of ESBL-PE exhibits significant global variation, with notably higher rates in developing countries compared to developed regions. Country-level meta-analyses showed a high ESBL-PE prevalence across developing countries. For instance, ESBL prevalence was 40% in Pakistan [10], 49% in Ethiopia [11], 34.6% in Nigeria [12], 29% in Nepal [13], and 42% in East African hospitals [14]. In contrast, prevalence rates in developed countries like Germany and the UK reach up to 10% [15, 16]. In Australia, the “Australian Group on Antimicrobial Resistance” reported a prevalence of 7.7% for the ESBL phenotype in *E. coli* isolates from bacteremia cases in 2021 [17]. This disparity in ESBL-PE rates between developed and developing countries may be attributed to several factors. Developing countries face high levels of antibiotic misuse, insufficient monitoring, widespread of antibiotic self-medication, incomplete treatment courses, and overprescription by healthcare providers [18, 19]. In Egypt, the availability of antibiotics without a prescription in community pharmacies has led to widespread of antibiotic self-medication and misuse [20–22]. Furthermore, a significant percentage of hospitals (38.2%) lack an ASP (Antimicrobial Stewardship Program) [23]. Even in hospitals with ASPs, healthcare workers encounter numerous challenges, including inadequate infection control programs, high workloads, limited resources, insufficient training in infection control, and staff shortages, all of which hinder adherence to standard precautions [24].

In response to the absence of a surveillance system for ESBL-PE infections in Egypt and to bridge the significant knowledge gap in this area, this meta-analysis was conducted. By synthesizing existing literature, we aimed to quantify the prevalence of ESBL-PE infections and elucidate the molecular dissemination of ESBL genes. We further stratified this prevalence by acquisition source (community-acquired vs. nosocomial) and bacterial species. Additionally, we aimed to uncover temporal trends to assess the spread over time. The findings of this research provide a thorough understanding of the epidemiology of ESBL-PE in Egypt. These insights are invaluable for guiding policy development, enhancing antimicrobial stewardship programs, and supporting infection control strategies.

## Methods

### Search strategy

A comprehensive literature search was conducted across the following databases: PubMed, Scopus, Google Scholar, Web of Science, and the Egyptian Knowledge Bank, covering the period from January 1, 2010, to July 2, 2024. We selected studies published between 2010 and 2024 to ensure the inclusion of the most up-to-date data, reflecting current trends in the prevalence of ESBL-PE in Egypt. This timeframe also allows for a comprehensive analysis of trends over a significant period while maintaining the relevance of the findings to current public health concerns. The search was conducted using the following keywords and Boolean operators: (“Extended-spectrum beta-lactamases” or ESBL) and (Enterobacteriaceae or “Enteric bacteria” or “Gram-negative bacteria” or “Gram-negative rods” or Enterobacterales or “*E. coli*” or “*Klebsiella*” or *Enterobacter* or *Proteus* or *Serratia* or *Citrobacter*) and Egypt*. The search strategy was modified to meet the specific requirements of each database used. Detailed search strategies for each database are provided in Table S1. For this study, we initially developed a protocol for the systematic review; however, it was not registered. Nevertheless, we adhered strictly to the original protocol throughout the review process, with no modifications made to the search strategy, eligibility criteria, or other prespecified analyses. This study followed the PRISMA (Preferred Reporting Items for Systematic Reviews and Meta-Analyses) guidelines [25]. Tables S2 and S3 present the PRISMA 2020 for Abstracts Checklist and the PRISMA Main Checklist (27-item checklist), respectively.

### Eligibility criteria

Studies were included if they met the following criteria: (1) primary studies reporting the prevalence of ESBL-PE in Egypt; (2) utilization of valid phenotypic detection methods of ESBL; (3) clinical specimens collected from patients; and (4) studies published between 2010 and 2024. In this study, nosocomial infections are defined as infections acquired during hospital care that occur more than 48 h after admission and were not present at the time of admission [26, 27]. In contrast, community-acquired infections are those contracted outside the hospital or infections that become clinically evident within 48 h of hospital admission [28]. Studies were excluded if they met any of the following criteria: not conducted in Egypt, involved specimens from food, animals, or healthy individuals for screening purposes (e.g., nasal or rectal swabs for carriage detection in asymptomatic individuals), or included samples partially or entirely selected from multidrug-resistant bacteria. Additionally, case reports, reviews, and conference abstracts were excluded. The study selection was conducted by two independent authors according to the predefined inclusion and exclusion criteria that were cross-checked by the other two. Any disagreements were resolved through consensus among all authors.

### Data extraction

Data extraction was carried out independently by two investigators and then cross-verified by two others. For each included study, the following information was extracted: first author’s last name, study period, location, total sample size, bacterial species tested, number of ESBL-positive isolates, type of infection (community vs. nosocomial), specimen type, phenotypic method for ESBL detection, and the prevalence of ESBL genes.

### Quality assessment

The quality of the included studies was rigorously assessed by two independent reviewers employing the “Joanna Briggs Critical Appraisal Checklist for Prevalence Studies” [29]. While the original questions from the checklist were retained, slight wording adjustments were made to align with the specific objectives of our study. Additionally, question 9, “Was the response rate adequate, and if not, was the low response rate managed appropriately?” was omitted, as it was not applicable to our study. The original checklist items are outlined in Table S4, with a cutoff score of 5 out of 8 established to denote that a study meets the threshold for fair quality. This checklist systematically evaluated several critical aspects: the appropriateness of the sampling frame in capturing the target population, the adequacy of the sampling methods in accurately reflecting the true distribution of isolates, and the sufficiency of the sample size. Furthermore, the checklist scrutinized whether the study subjects and settings were comprehensively described, ensuring that the data analysis was conducted with adequate coverage of the identified sample. It also assessed the use of validated methods for the identification of ESBL-PE, the reliability of ESBL measurement using standardized methods, and the appropriateness of the statistical analysis employed to assess prevalence.

### Data synthesis

A meta-analysis of proportions was conducted using the meta package in the R programming language. The pooled prevalence with 95% confidence intervals (CI) was calculated using a random effects framework employing the inverse variance method. Subgroup analyses were performed based on the type of infection (community vs. nosocomial), bacterial species, and the duration of the studies. To ensure a more reliable meta-analysis, we only included studies that provided at least three different data points or estimates. Heterogeneity was assessed using I-squared (I²) and Cochran’s Q statistics to evaluate the variation between studies. I² values above 75% were considered indicative of high heterogeneity [30]. Sensitivity analyses were performed using a leave-one-out method to assess the robustness of the findings. Publication bias was assessed using a funnel plot and Egger’s test, with a *P*-value of less than 0.05 indicating evidence of publication bias.

## Results

### Characteristics of the included studies

**Fig. 1:**
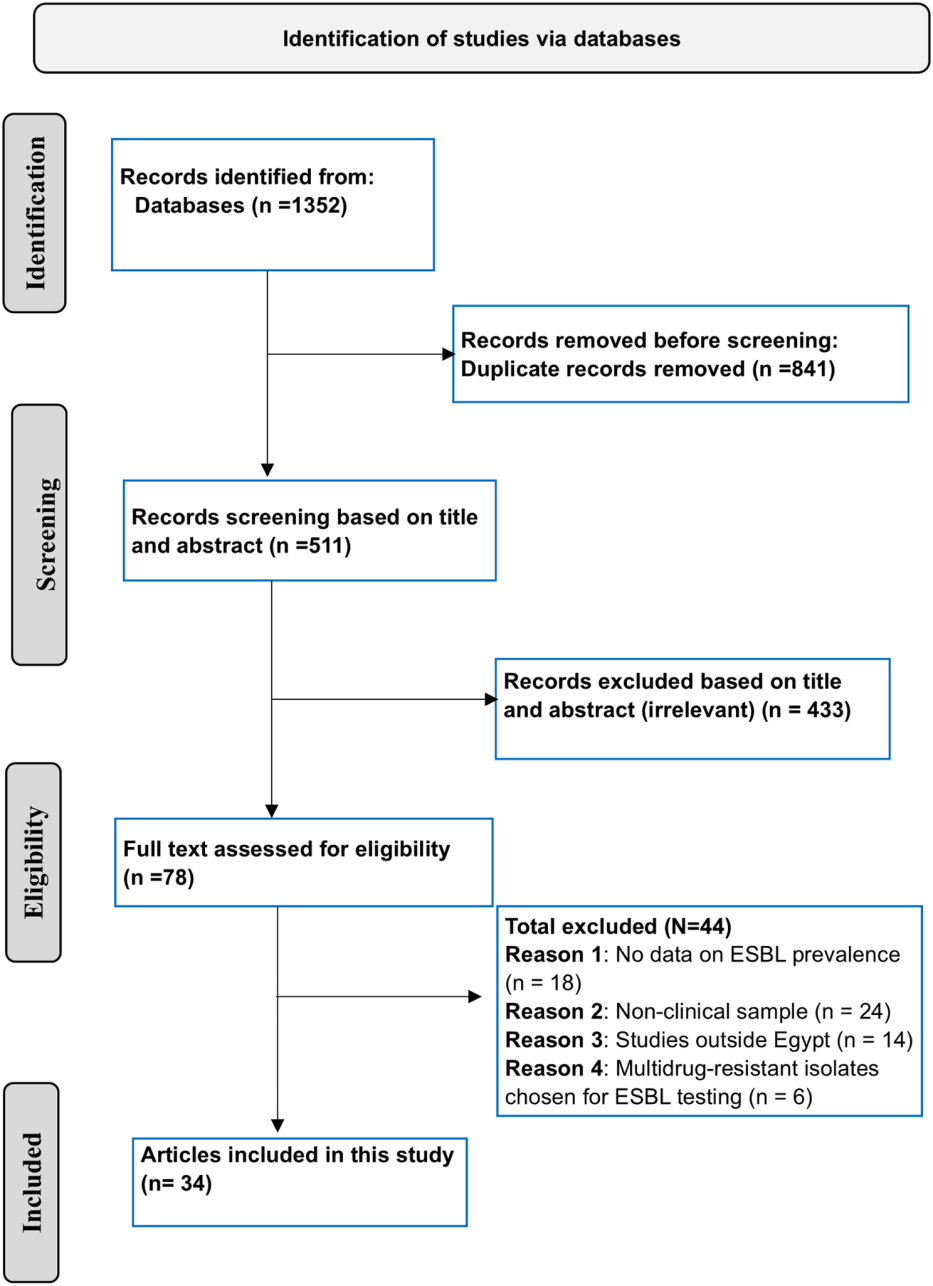
PRISMA flowchart depicting the study selection process for inclusion

**Fig. 2:**
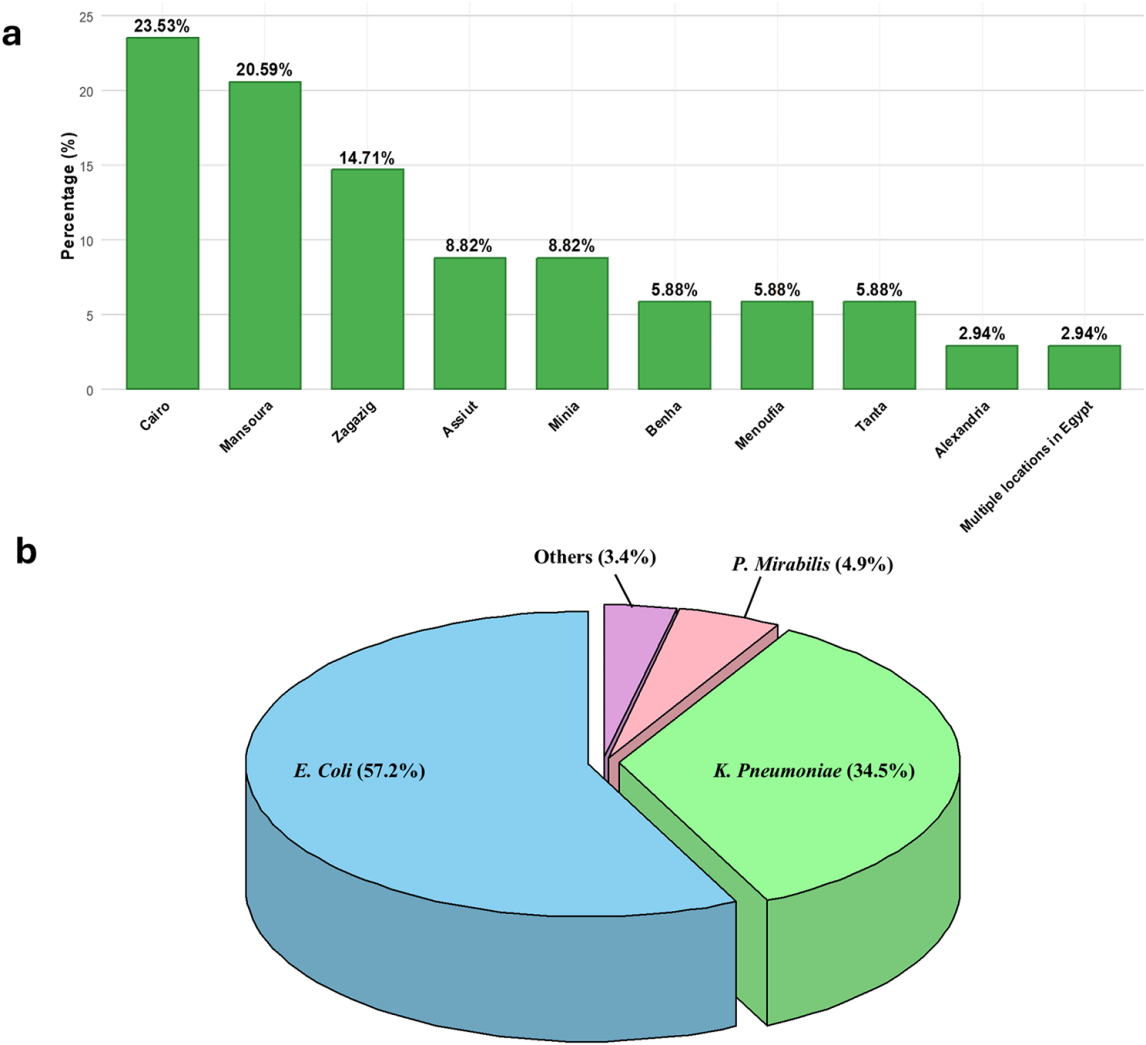
Percentages of geographical distribution and tested *Enterobacteriaceae* species in the included studies. (**a**) Geographical distribution of studies. (**b**) Distribution of tested *Enterobacteriaceae* species. The “Others” category includes *Enterobacter*, *Serratia*, and *Citrobacter* species

The detailed characteristics of the included studies are presented in Table 1. A total of 34 studies, involving 4,528 isolates, were included in this meta-analysis [31–64], as shown in Fig. 1. The periods of the included studies ranged from 2007 to 2023. Among the studies, 10 reported ESBL-PE prevalence in both community and hospital-acquired infections [35, 37, 39, 43, 54, 55, 57–59, 61]. Four focused exclusively on community-acquired infections [42, 51, 56, 63] and 9 were specific to hospital-acquired [32, 33, 36, 41, 45, 47, 48, 53, 64]. Additionally, 11 studies were hospital-based but did not specify the source of infection [31, 34, 38, 40, 44, 46, 49, 50, 52, 60, 62]. Among the studies, 13 focused on *E. coli* [33, 35–37, 39, 40, 42, 48, 51, 57, 59, 62, 63], while five concentrated on *K. pneumoniae* [38, 43, 46, 54, 60]. One study examined both *E. coli* and *Proteus mirabilis* [34], and two focused exclusively on *Proteus mirabilis* [50, 58]. Additionally, five studies explored both *E. coli* and *K. pneumoniae* [31, 41, 49, 55, 61]. The remaining studies examined a broader spectrum of *Enterobacteriaceae* species [16, 28, 29, 31, 36, 37, 40, 47]. Most of the studies were conducted in Cairo, accounting for 23.53%, with *E. coli* representing the majority of tested *Enterobacteriaceae* species at 57.2%. Figure 2 shows the geographical distribution of the included studies and the distribution of tested *Enterobacteriaceae* species. In terms of quality assessment, all the studies included achieved a score above 5, which we consider meeting at least a fair standard of quality, as shown in Table S5.

**Table 1:** Characteristics of the included studies

Last Name of First Author [citation]	Study period	Location	Total Sample Size	Bacteria Tested	ESBL positive	Type of infection (Community Vs. Nosocomial) *	Specimen	Phenotypic Detection OF ESBL
Khater, 2014 [59]	2012–2013	Benha	45	*E. coli*	24	Both	Urine	CDT
Shaaban, 2022 [50]	2021	Cairo	58	*Proteus*	30	NS	Urine, Wound, Blood, Sputum, CSF	DDST
Ragab, 2024 [39]	NS	Benha	41	*E. coli*	34	Both	Urine	Disk Diffusion, DDST
Masoud, 2022 [40]	NS	Minia	60	*E. coli*	37	NS	Urine	CDT
Essawy, 2018 [37]	2017	Tanta	44	*E. coli*	37	Both	Urine	DDST, MDDST, ESBL E-Test
ElTaweel, 2024 [34]	2021–2022	Mansoura	66	*Proteus mirabilis*	38	NS	Various Clinical Sources	CDT
Gaballah, 2023 [38]	NS	Alexandria	50	*K. pneumoniae*	39	NS	Various Clinical Specimens	Disk Diffusion, DDST
Abd El-Hamid, 2010 [32]	2009–2010	Zagazig	68	*E. coli*,* K. pneumoniae*,* Enterobacter*,* Proteus*,* Serratia*,* Citrobacter*	45	Nosocomial	Blood, Urine, CSF, Endotracheal Tube Aspirates.	DDST
Abdallah, 2015 [53]	2013	Zagazig	94	*E. coli*,* K. pneumoniae*,* Enterobacter*,* Citrobacter*,* Serratia*,* Proteus*	46	Nosocomial	Blood	Vitek 2 System, CDT
Abdel-Moaty, 2016 [57]	2012	Cairo	90	*E. coli*	47	Both	Mostly urine	CDT
Rashwan, 2023 [42]	2022–2023	Assiut	73	*E. coli*	47	Community	Stool	Vitek 2 System
Zaki, 2019 [48]	2016–2018	Mansoura	88	*E. coli*	49	Nosocomial	Blood	DDST, CDT
Elsayed, 2024 [45]	2019–2020	Mansoura	105	*E. coli*,* K. pneumoniae*,* Enterobacter*,* Serratia*,* Proteus*	50	Nosocomial	Urine, Respiratory Tract Infections, Wound Infections, Blood	DDST
EL-Ganiny, 2016 [60]	NS	Zagazig	100	*K. pneumoniae*	50	NS	Surgical Wounds, Urinary Catheters, Burns, Blood, Sputum	DDST
El-Mahdy, 2015 [43]	2011–2012	Cairo	112	*K. pneumoniae*	52	Both	Sputum, Stool, Blood, Urine, Pus	DDST, CDT
Sharaf, 2024 [46]	NS	Cairo	115	*K. pneumoniae*	53	NS	Blood Culture, Endotracheal and Sputum Culture, Urine, Wound Swabs	Disk Diffusion, CDT
Al-Sayed, 2018 [63]	2016–2017	Zagazig	112	*E. coli*	56	Community	Urine	DDST
Essam, 2011 [31]	NS	Mansoura	86	*E. coli*,* K. pneumoniae*	60	NS	Urine	DDST
Abd El-Aziz, 2021 [44]	NS	Mansoura	80	*E. coli*,* K. pneumoniae*,* Enterobacter*	67	NS	Urine, Sputum	MDDST
Makled, 2016 [64]	2013–2015	Menoufia	120	*E. coli*,* K. pneumoniae*,* Enterobacter*,* Proteus*,* Citrobacter*,* Serratia*	67	Nosocomial	Urine	Disk Diffusion, CDT
Alshaikh [62]	2021–2022	Tanta	100	*E. coli*	67	NS	Urine	DDST
Thabit, 2011 [35]	2009	Assiut	136	*E. coli*	72	Both	Urine	CDT, DDST, ESBL-E-Test
El Maghraby, 2024 [36]	2020–2022	Zagazig	128	*E. coli*	73	Nosocomial	Urine	CDT
Rizk, 2022 [33]	2021	Mansoura	100	*E. coli*	74	Nosocomial	Urine	Disk Diffusion, DDST
Hassuna, 2020 [51]	2016–2018	Minia	134	*E. coli*	80	Community	Urine	MDDST, CHROMagar ESBL
Fam, 2010 [61]	2007–2008	Cairo	520	*E. coli*,* K. pneumoniae*	83	Both	Mostly Urine	DDST
Khalifa, 2019 [47]	2014	Multiple locations in Egypt	126	*E. coli*,* K. pneumoniae*,* Serratia*,* Salmonella*,* Morganella morganii*	95	Nosocomial	Blood, Urine, Respiratory Sputum, Pus	Disk Diffusion
El-Shalakany, 2014 [52]	NS	Menoufia	160	*E. coli*,* K. pneumoniae*,* Enterobacter*,* Citrobacter*,* Serratia*,* Proteus*	97	NS	Urine, Sputum, Stool, Blood, Pus	Disk Diffusion, PCDDT
Shash, 2019 [55]	2016–2017	Cairo	250	*E. coli*,* K. pneumoniae*	100	Both	Urine	Disk Diffusion, DDST, CDT
Ibrahim, 2014 [54]	2014	Assiut	143	*K. pneumoniae*	108	Both	Urine	DDST, CDT, ESBL-E-Test
Amer, 2019 [49]	2016–2018	Cairo	168	*E. coli*,* K. pneumoniae*	113	NS	Urine, Pus, Blood, Sputum, Semen, Stool	DDST, CDT
Mohamed, 2020 [56]	2018	Minia	440	*E. coli*,* K. pneumoniae*,* Enterobacter*,* Citrobacter*,* Proteus*	311	Community	Urine	Disk Diffusion, DDST
El Kholy, 2011 [41]	2008–2010	Cairo	456	*E. coli*,* K. pneumoniae*	326	Nosocomial	Blood, Urine, Sputum, Pus	DDST, CDT
Salama, 2021 [58]	2016–2017	Mansoura	60	*Proteus mirabilis*	17	Both	Urine	MDDST

CDT: Combination Disk Test; DDST: Double Disk Synergy Test; MDDST: Modified Double Disk Synergy Test; ESBL E-Test: Extended Spectrum Beta-Lactamase E-Test; PCDDT: Phenotypic Confirmatory Disk Diffusion Test, NS: Not specified

* All the unspecified studies were hospital-based, but the source of infection, whether community-acquired or hospital-acquired, was not specified

### Prevalence of ESBL-producing *Enterobacteriaceae*

**Fig. 3:**
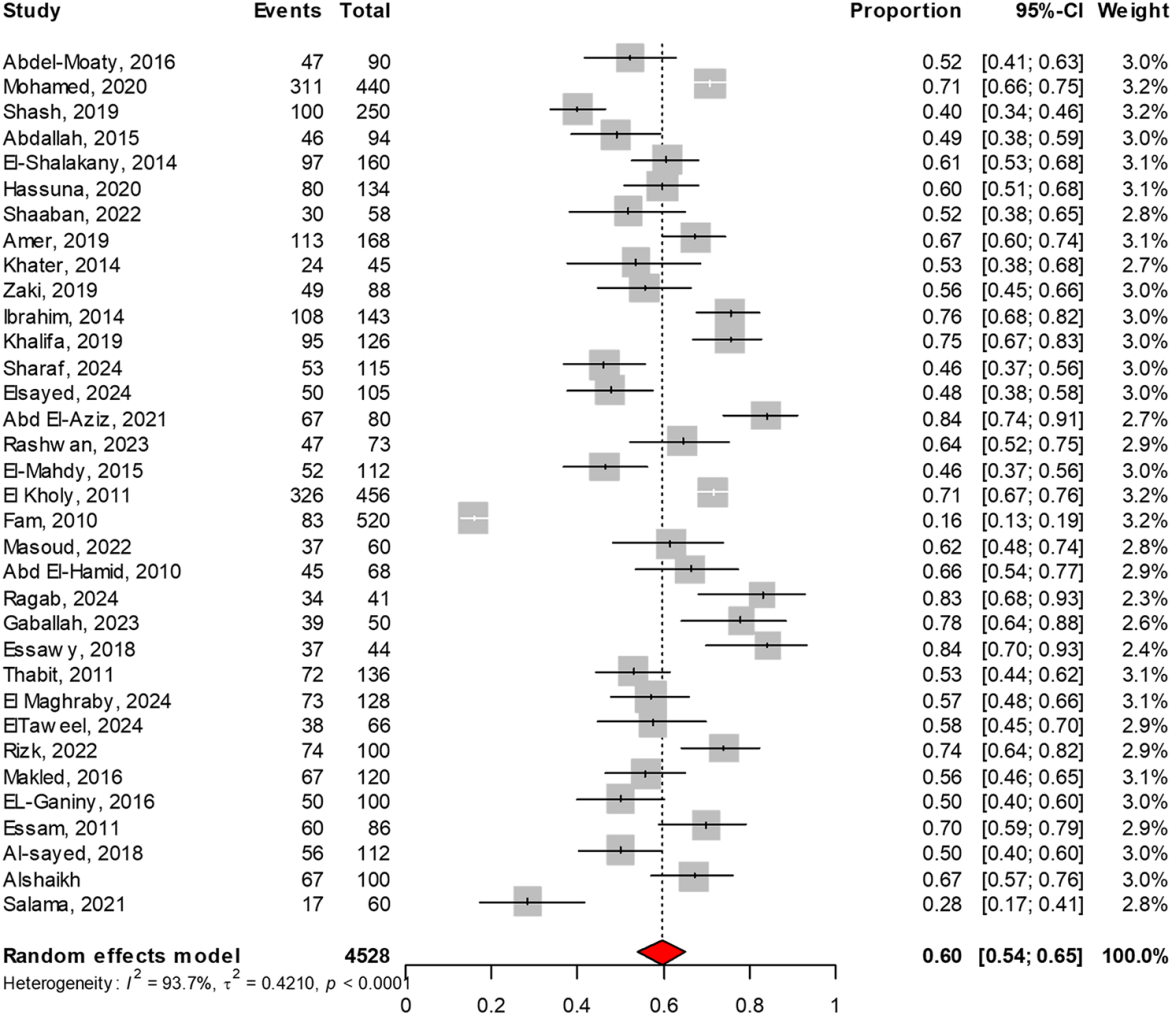
Meta-analysis of the prevalence of ESBL-producing *Enterobacteriaceae* in Egypt. This meta-analysis, encompassing data from 34 studies with a combined sample size of 4,528 isolates, estimates the prevalence to be 60% (95% CI: 54–65) based on random effects models

**Fig. 4:**
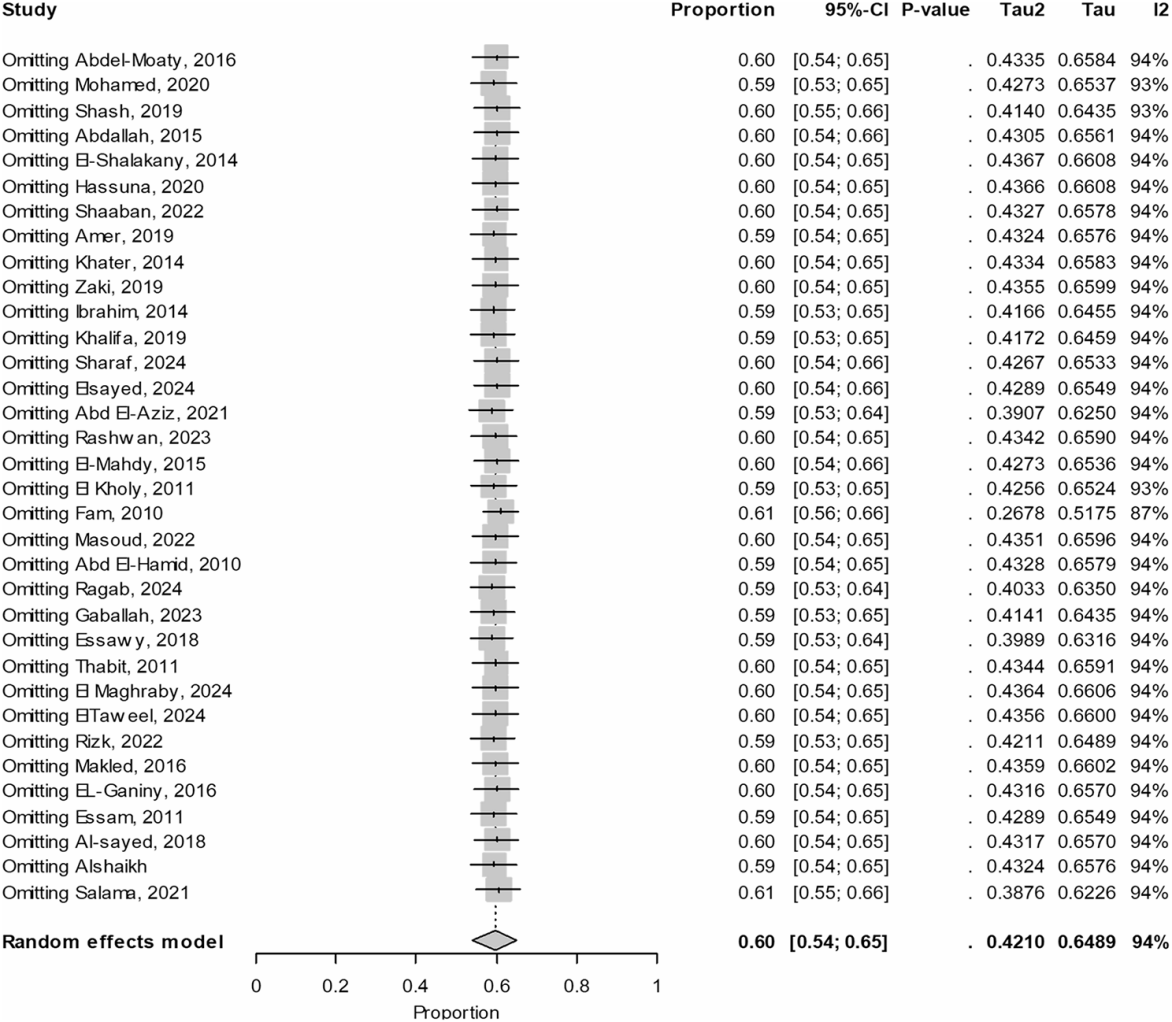
Sensitivity analysis using the leave-one-out method. The analysis reveals that the overall effect estimate remains stable, with changes of no more than 1%, indicating the robustness of the meta-analysis

**Fig. 5:**
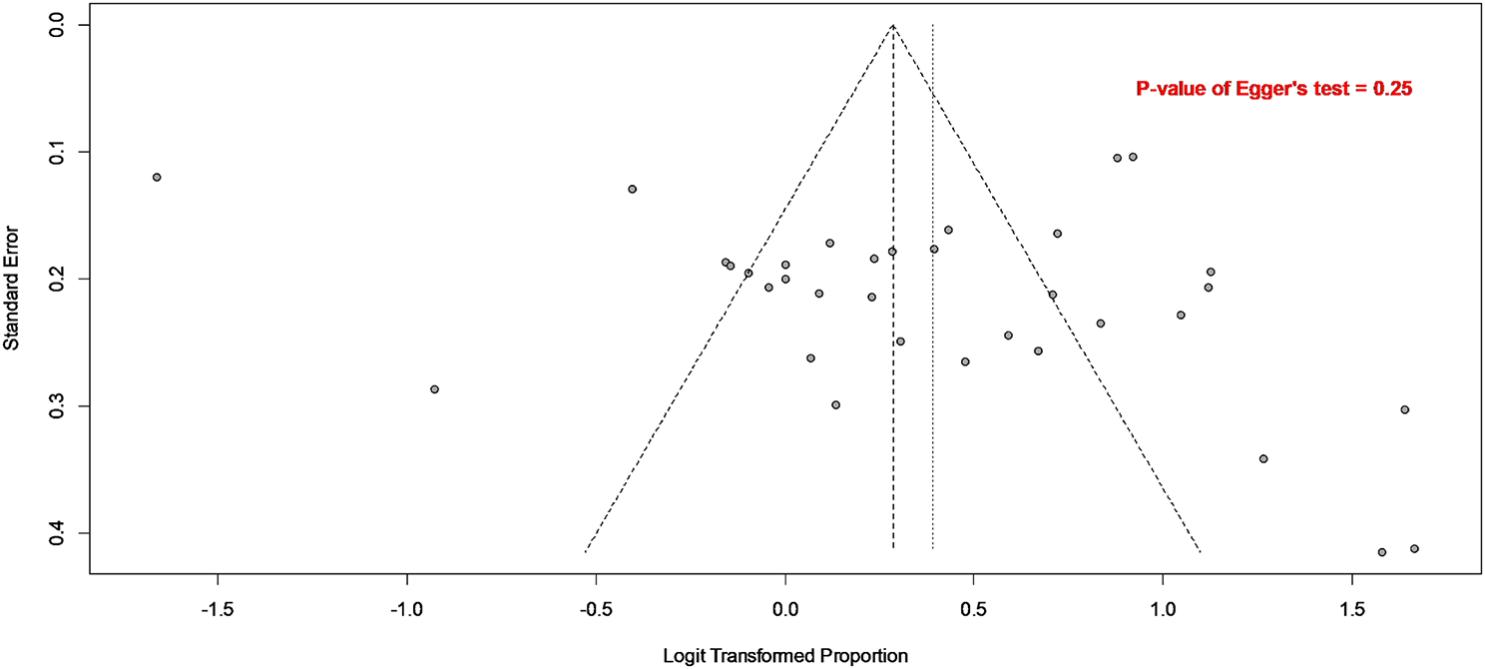
Funnel plot for publication bias testing. The absence of publication bias is indicated by the p-value from Egger’s test (*P* = 0.25)

Thirty-four studies were included in this meta-analysis with a total sample size of 4,528, revealing that the prevalence of ESBL production among *Enterobacteriaceae* in Egypt was 60% (95% CI: 54–65, I² =94%), as shown in Fig. 3. Systematically removing one study at a time in the leave-one-out meta-analysis revealed that the overall prevalence remained stable, with changes of no more than 1%. This suggests that the meta-analysis is robust, as illustrated in Fig. 4. Moreover, the *P*-value from Egger’s test (0.25), as presented in Fig. 5, indicates the absence of publication bias.

### Prevalence of ESBL-producing *Enterobacteriaceae* in community-acquired and nosocomial infections

**Fig. 6:**
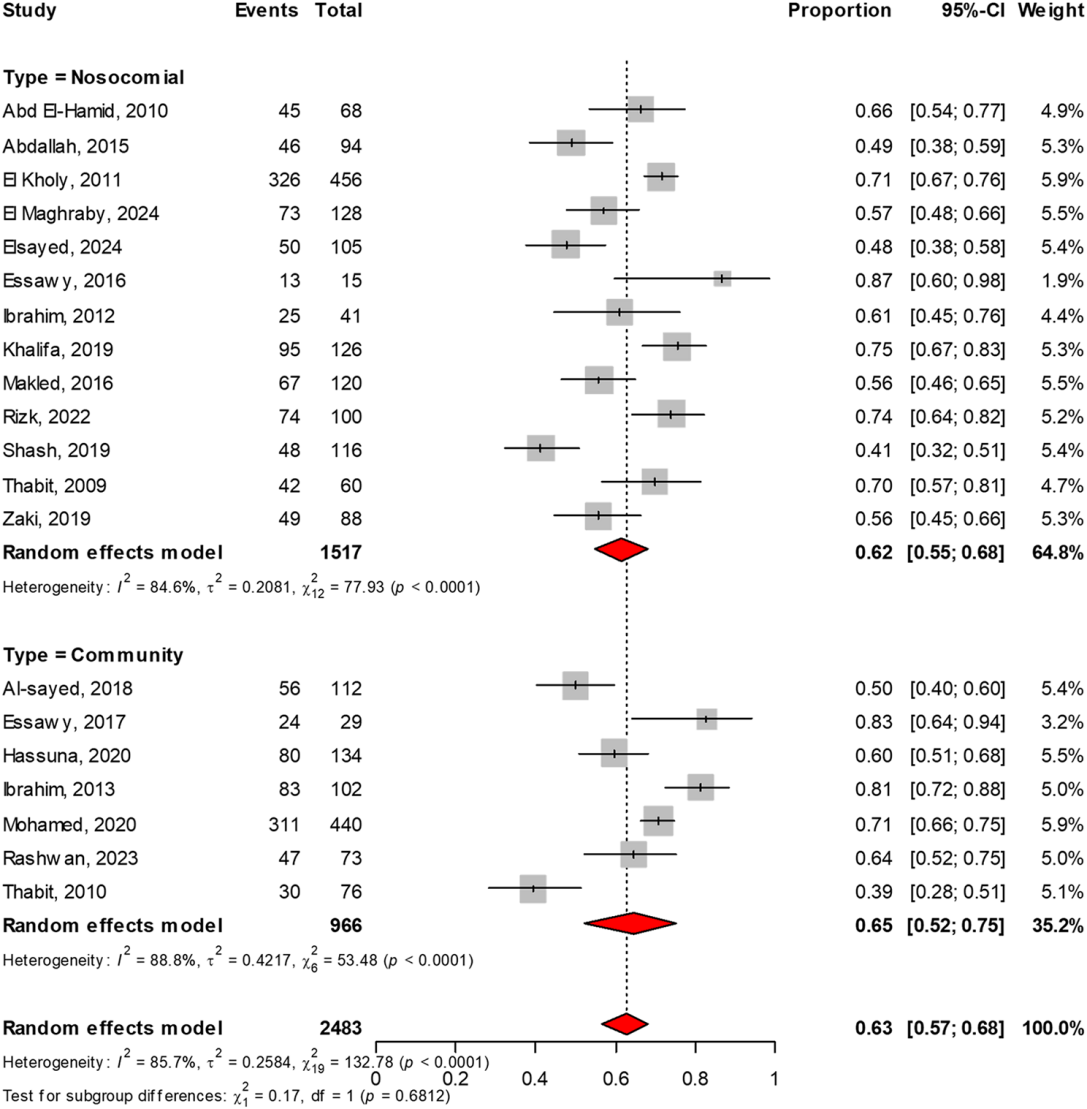
Meta-analysis of the prevalence of ESBL-producing *Enterobacteriaceae*, stratified by infection source (nosocomial versus community-acquired infections). The analysis indicates no statistically significant difference between the two sources, with a *P*-value of 0.68

Thirteen studies, with a total sample size of 1,517, reported on the prevalence of ESBL-PE in nosocomial infections. The meta-analysis of these studies revealed a prevalence rate of 62% (95% CI: 55–68, I² =85%). In contrast, seven studies, encompassing a sample size of 966, focused on the prevalence of ESBL-PE in community infections. The meta-analysis indicated a prevalence rate of 65% (95% CI: 52–75, I² =89%). There was no significant difference in the prevalence of ESBL-PE between nosocomial and community infections, as evidenced by a *P*-value of 0.68, as illustrated in Fig. 6.

### Prevalence of ESBL-producing *Enterobacteriaceae* by bacterial species

We conducted a meta-analysis to assess the prevalence of ESBL-producing bacteria among three *Enterobacteriaceae* species: *E. coli*,* K. pneumoniae*,* and Proteus mirabilis*, each with at least three estimates available. The prevalence of ESBL-producing *E. coli* was 64% (95% CI: 58–70, I²=85%), which was comparable to *K. pneumoniae*, showing a prevalence of 63% (95% CI: 54–71, I²=84%). In contrast, *Proteus mirabilis* exhibited a slightly lower prevalence of 46% (95% CI: 32–60, I²=74%). There was no significant difference in the prevalence of ESBL among the three species, as evidenced by a *P*-value of 0.06, as shown in Fig. S1.

### Temporal trends in ESBL-producing *Enterobacteriaceae*

**Fig. 7:**
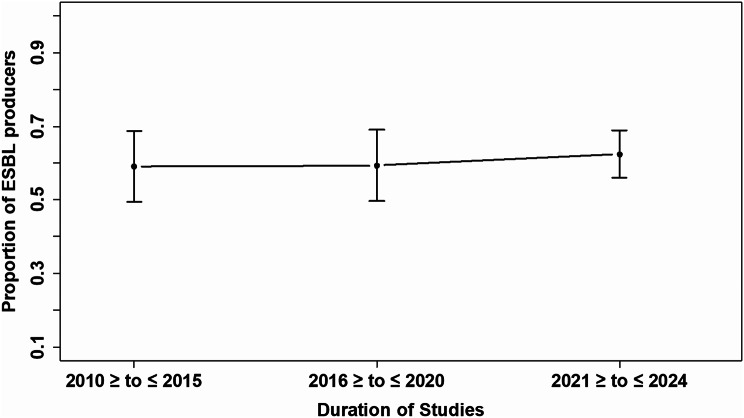
Temporal trends in the prevalence of ESBL-producing *Enterobacteriaceae*. Each solid dot represents the pooled prevalence of ESBL-producing *Enterobacteriaceae* for the periods 2010–2015, 2016–2020, and 2021–2024, with vertical error bars indicating the 95% confidence intervals. The analysis shows a consistent prevalence rate from 2010 to 2024, with a slight, statistically non-significant increase after 2021 (*P* = 0.81)

The temporal trend analysis of the prevalence of ESBL-PE over different study periods indicates that the prevalence has remained relatively stable over time. From 2010 to 2015, the pooled prevalence was 59% (95% CI: 49–68). This trend continued with a similar prevalence observed from 2016 to 2020, where the pooled prevalence was 59% (95% CI: 49–69). In the period from 2021 and beyond, there was a slight non-significant increase in prevalence to 62% (95% CI: 56–69), *P* = 0.81, as illustrated in Fig. 7.

### Molecular characterization of ESBL genes in *Enterobacteriaceae*

Among phenotypically confirmed ESBL-producing *E. coli*, *bla*_CTX−M_ exhibits the highest pooled prevalence at 73% (95% CI: 55–86). In contrast, the *bla*_TEM_ gene shows a slightly lower pooled prevalence of 60% (95% CI: 33–82). The *bla*_SHV_ gene, however, has the lowest pooled prevalence at 22% (95% CI: 11–41). The differences in the prevalence of these genes were statistically significant, with a *P-*value of < 0.01 and the heterogeneity was substantial (I² > 90%), as shown in Fig. S2. Studies that further characterized of *bla*_CTX−M_, subtypes identified *bla*_CTX−M−15_ as the predominant variant [47, 51, 53, 55, 56]. Research on other *Enterobacteriaceae* species is limited and constrained by small sample sizes, thereby hindering the feasibility of conducting a robust meta-analysis.

## Discussion

Evaluating the prevalence of AMR is essential for playing a key role in public health surveillance by detecting emerging resistance trends and enabling timely interventions to prevent the spread of resistant pathogens. In recent decades, there has been a marked increase in the incidence and diversity of infections caused by ESBL-PE, with notable variation observed across different institutions and countries. In light of this growing concern, we meta-analyzed the prevalence of ESBL-PE in Egypt, synthesizing data from 34 studies and encompassing a total of 4,528 isolates. The overall prevalence was 60%, with no significant difference in the prevalence between hospital-acquired and community-acquired infections. Among phenotypically confirmed ESBL-producing *E. coli*, *bla*_CTX−M_ (73%) was the most prevalent, followed by *bla*_TEM_ and *bla*_SHV_. Additionally, temporal analysis showed stable ESBL prevalence over time. This alarmingly high prevalence of ESBL-PE underscores the urgent need for comprehensive public health strategies to effectively mitigate its spread.

Compared with other meta-analyses, the prevalence of ESBL production among *Enterobacteriaceae* in Egypt is notably high at 60% (95% CI: 54–65), surpassing rates reported in Nepal, Ethiopia, Nigeria, Pakistan and Eastern Africa [10–14]. Pakistan reported an overall pooled prevalence of 40% (95% CI: 34–47) [10], while in Ethiopia, it was 49% (95% CI: 39–60) [11]. Nigeria had a prevalence of 34.6% (95% CI: 26.8–42.3) [12]. In Nepal, the prevalence was 29% (95% CI: 26–32%) [13], and in East African hospitals, it was 42% (95% CI: 34–50) [14]. The results of our sensitivity analysis demonstrate that the overall pooled prevalence remained consistent with variations of no more than 1%, thereby affirming the robustness of our findings.

The spread of ESBL-PE is primarily driven by critical factors such as the improper use of antibiotics [65, 66] and deficiencies in infection control measures [55–57]. Therefore, the high prevalence of ESBL-PE in Egypt can be largely attributed to the availability of antibiotics without a prescription in community pharmacies, which has resulted in widespread self-medication and misuse [20–22], inadequate infection control programs [24, 67], and the significant presence of ESBL-PE in food and in animal products and livestock [68–70], which can subsequently spread to humans. Furthermore, a significant percentage of hospitals (38.2%) lack an ASP [23]. Even in hospitals with ASPs, healthcare workers face significant challenges, such as inadequate infection control programs, high workloads, limited resources, insufficient training, and staff shortages, which hinder adherence to standard precautions [24]. The similarly high prevalence of ESBL-PE in both hospital and community-acquired infections implies that these bacteria are actively circulating in the community and not solely confined to healthcare settings. This indicates that the factors driving the spread of ESBL-PE are widespread and present in both settings.

Concerning the molecular characterization of ESBL genes in *E. coli* in Egypt, a pooled analysis indicates that *bla*_CTX−M_ is the most prevalent, detected in 73% of confirmed ESBL-producing isolates, followed by *bla*_TEM_ at 60%. The *bla*_SHV_ gene exhibits the lowest prevalence, found in 20% of isolates (*P* < 0.01). In the same vein, a meta-analysis conducted on the molecular characterization of ESBL-PE in East, Central, and Southern Africa revealed that the *bla*_CTX−M_ gene, particularly the *bla*_CTX−M−15_ variant, is the most prevalent ESBL gene among the isolates studied [71]. Similarly, in Gulf Cooperation Council countries, the predominant genes responsible for ESBL resistance in *Enterobacteriaceae* isolates are *bla*_CTX−M_, followed closely by, or co-dominant with, *bla*_TEM_ and *bla*_SHV_ [72]. The majority of ESBLs are classified under Ambler class A, with the SHV, TEM, and CTX-M types being the most prevalent. Since the early 2000s, *E. coli* strains producing CTX-M enzymes have emerged as a significant etiological agent in community-acquired infections, particularly in cases of urinary tract infections [71]. Since then, the prevalence of *E. coli* harboring the *bla*_CTX−M_ gene has been increasing dramatically [71].

The susceptibility of ESBL-producing organisms to non-beta-lactam antibiotics varies widely across the included studies, reflecting both treatment challenges and possible therapeutic options. Overall, high resistance to cotrimoxazole, ciprofloxacin, and gentamicin was consistently observed among ESBL-producing isolates, while imipenem remains highly effective across studies [43, 48, 50, 56, 57, 63]. For cotrimoxazole, ESBL producers showed significantly high resistance rates. Abdel-Moaty et al. observed an 89% resistance rate compared to 67% in non-producers [57]. Similarly, Al-Sayed et al. observed 67.8% resistance in ESBL producers compared to 35.7% in non-producers [63]. Zaki et al. further noted a 32.7% resistance rate in ESBL-producing *E. coli* [48]. Ciprofloxacin also showed a high resistance pattern, with Shaaban et al., reporting a 33.3% resistance rate in ESBL producers versus 17.9% in non-producers [50]; this aligns with Abdel-Moaty et al. [57] and El-Mahdy et al. [43] who observed even higher resistance rates of 85% and 79% in ESBL producers respectively. Similarly, gentamicin resistance was also elevated in ESBL producers, as indicated by El-Mahdy et al. [43] and Mohamed et al. [56] with rates of 90% and 73%, respectively, though Zaki et al. [48] reported a somewhat lower rate of 40.8%. In contrast, imipenem showed high effectiveness against ESBL-producing strains. Shaaban et al. [50] and Mohamed et al. [56] reported complete susceptibility, and Al-Sayed et al. [63] found only minimal resistance (1.7%) without significant differences from non-producers. This trend highlights imipenem’s potential as a reliable treatment for ESBL-producing infections, in contrast to the high resistance seen with other non-beta-lactams. The difference in co-resistance to non-beta-lactam antibiotics among ESBL producers in community and hospital-acquired infections was examined in one study, which found variations in co-resistance [55]. However, these variations were not statistically significant except for nitrofurantoin. All ESBL producers in community-acquired infections were susceptible to nitrofurantoin, while only 58.3% of hospital-acquired UTI cases with ESBL producers showed susceptibility (*p* < 0.01) [55]. These findings underscore the necessity for ongoing research to better understand the mechanisms driving co-resistance among ESBL producers in different settings, as this knowledge can significantly impact the effectiveness of treatment options. Additionally, one study examined the molecular mechanisms of co-resistance to non-beta-lactam antibiotics among ESBL producers [47]. Of the isolates tested, 165 (53%) were found to carry the *aac(6’)-Ib-cr* gene, which confers resistance to amikacin and ciprofloxacin. Additionally, a statistically significant association was observed between the *aac(6’)-Ib-cr* gene and *bla*_CTX−M_ genes (*p* < 0.01) [47]. This raises concerns about treatment options for infections caused by these resistant strains and underscores the need for further studies to explore the mechanisms of co-resistance in greater depth.

To combat the spread of ESBL-PE, a comprehensive approach is necessary. Antibiotic stewardship programs in hospitals and communities should ensure proper antibiotic use, supported by public health campaigns and education. Infection control measures, such as enhanced hygiene practices and patient isolation, are crucial in both settings. Robust surveillance systems and data sharing can track and manage resistance trends. Public education should target misconceptions about antibiotics, and stricter regulations on prescriptions and agricultural antibiotic use are needed to reduce misuse and the spread of resistance.

### Strength and limitation

A key strength of this analysis is the inclusion of a large number of studies and the stratification of the analysis based on the source of infection, which is crucial for accurate interpretation. Moreover, the sensitivity analysis further indicates that the estimates are robust, as there are no influential outliers deviating the pooled prevalence by more than 1%. Additionally, there is no evidence of publication bias, as evidenced by the *P*-value of Egger’s test (0.25). Nevertheless, we acknowledge the following limitations in this study. First, the absence of prevalence data from certain regions in Egypt restricts the comprehensiveness and potentially the generalizability of our findings. Second, the ability to stratify the dissemination of ESBL genes based on the source of (community vs. nosocomial) was constrained by the limited availability of studies providing such specific data. Finally, high heterogeneity was observed in this meta-analysis, which is inherent and commonly expected in meta-analyses of prevalence data [73, 74]. This high heterogeneity is primarily due to large sample sizes in proportional data, which produce very precise estimates and result in narrow CIs. Consequently, even small differences in prevalence between studies can lead to minimal overlap between these CIs, thereby increasing heterogeneity [73]. In addition to this inherent heterogeneity typical in meta-analyses of prevalence, variability may also be attributable to regional and institutional differences in antibiotic use and stewardship practices. A previous study highlighted an institutional disparity in antibiotic stewardship practices, with 38.2% of hospitals lacking an ASP [23]. This heterogeneity was particularly evident in our analysis of *bla* genes among phenotypically confirmed ESBL-producing *E. coli*, where broader CIs contributed to greater variability in pooled estimates. Historically, the *bla*_TEM_ and *bla*_SHV_ genes were the most prevalent ESBL genes worldwide [9]. In recent years, however, *bla*_CTX−M_ has emerged as the dominant ESBL family globally [9], including in Egypt, where it is now the most prevalent. This shift has not been consistent across all Egyptian regions. Among the thirteen included studies in that analysis, Zaki et al. reported that the *bla*_SHV_ gene was the most common, followed by *bla*_TEM_ and *bla*_CTX−M_ [48]. Additionally, El Mahdi et al. and Hassuna et al. showed that *bla*_TEM_ was the most prevalent gene, followed by *bla*_CTX−M_ and *bla*_SHV_ [43, 51]. These regional differences contribute to heterogeneity in the meta-analysis, adding to the inherent variability typical of prevalence studies. Overall,

*bla*_CTX−M_ is the most prevalent ESBL gene family in most studies. This observed heterogeneity highlights the need for ongoing research to monitor these genes over time and better understand changing trends. These limitations highlight the imperative for further research to more thoroughly address these knowledge gaps.

## Conclusions

The prevalence of ESBL-PE in Egypt is alarmingly high at 60%. The comparable prevalence of ESBL-PE in both hospital-acquired and community-acquired infections indicates that these bacteria are not confined to healthcare settings but are actively circulating within the community. Consequently, there is a critical need for comprehensive public health strategies that effectively address the spread of ESBL-PE across both healthcare and community settings.

## Electronic supplementary material

Below is the link to the electronic supplementary material.

Supplementary Material 1

## Data Availability

All data generated and analyzed throughout this study were included either in this article or its supplementary information file.

## Abbreviations

ESBL-PE: Extended-Spectrum Beta-Lactamase-Producing Enterobacteriaceae
CI: Confidence Interval
WHO: World Health Organization
AMR: Antimicrobial Resistance
SBL: Serine Beta-Lactamase
MBL: Metallo-Beta-Lactamase
ASP: Antimicrobial Stewardship Program
PRISMA: Preferred Reporting Items for Systematic Reviews and Meta-Analyses
CDT: Combination Disk Test
DDST: Double Disk Synergy Test
MDDST: Modified Double Disk Synergy Test
PCDDT: Phenotypic Confirmatory Disk Diffusion Test
